# New Phenotypic Feature in a Patient With a Rare Triplication of the 22q11.2 Region Presenting With Peters Anomaly, Congenital Heart Disease, and Global Developmental Delay: A Case Report and Literature Review

**DOI:** 10.7759/cureus.26071

**Published:** 2022-06-18

**Authors:** Isra Idris, Abinash Pandey, Abdalaziz M Awadelkarim, Eltaib A Saad, Marsha Medows

**Affiliations:** 1 Pediatrics, NYC Health + Hospitals/Woodhull, New York City, USA; 2 Internal Medicine, Wayne State University Detroit Medical Center, Detroit, USA; 3 Internal Medicine, Saint Francis Hospital, Evanston, USA; 4 Pediatrics, New York University Grossman School of Medicine, New York City, USA

**Keywords:** peter's anamoly, subaortic stenosis, 22q11.2 deletion, triplication of 22q11.2 chromosome region, global developmental delay

## Abstract

The vulnerability of chromosome 22q11.2 region to rearrangement is due to several low copy repeat (LCR) sequences. These rearrangements are involved in syndromes that share similar phenotypic features. The rearrangements of the 22q11.2 chromosomal region are common, specifically, duplications and deletions associated with congenital anomalies and developmental disabilities disorders. However, the features associated with this chromosomal rearrangement remain largely unknown. We present, to the best of our knowledge, the third patient affected by triplication of the 22q11.2 chromosome region, who presents with Peters anomaly, global developmental delay, patent ductus arteriosus, and subaortic stenosis. This case highlights a new phenotypic feature associated with triplication of this genomic region.

## Introduction

The 22q11.2 deletion syndrome is due to the 22q11.2 chromosomal region, including phenotype previously known as DiGeorge/Velocardiofacial syndrome (DGS/VCFS), which has a prevalence estimated between 1:2000-1:17000 live births [1]. The rearrangements of the 22q11.2 chromosomal region are associated with multiple congenital anomalies, especially deletions and duplications. About 10% of these deletions are inherited from an affected parent, while 90% are de novo mutations [2]. The deletion of the 22q11 chromosomal region phenotype is variable. It includes the following features: velopharyngeal insufficiency, characteristic craniofacial, cardiovascular anomalies, palatal abnormalities, nasal voice, immune deficiency related to hypoplasia of the thymus, endocrine dysfunctions, such as hypocalcemia, a varying degree of cognitive defects, and intellectual disability [3].

Deletions are largely undetected. However, researchers suggested that the prevalence of duplications is about half of the deletions. Lately, 22q11.2 microduplication syndrome has been reported as a new genomic duplication syndrome. Additionally, it is phenotypically highly variable in appearance ranging from a normal or a mild learning disability to multiple congenital defects such as velopharyngeal insufficiency with and without cleft palate, heart defects, growth delay, and mild dysmorphic features [4].

Triplication (four copies) is a rare mutation; however, exploring the related phenotype may explain the dosage sensitivity of the genes in this chromosomal interval. We describe a patient with 646 Kb triplication of 22q11.2 (LCR22B-D) of the DiGeorge region. The triplication of the 22q11.2 region has been reported only twice earlier [3,4]. This genetic variant only causes a mild phenotype, such as learning difficulties, speech delay, and dysmorphic features (broad nasal bridge, bulbous nasal tip, epicanthal folds, and hand/foot abnormality. Furthermore, the triplication of this region could result in a wide range of phenotypic features ranging from mild to severe neurodevelopmental disorder, cardiac anomalies, and facial dysmorphisms. However, it may have varied features, some of which can also be seen in healthy individuals in the general population.

## Case presentation

We present a case of a 28-month-old female child who was born to a 34-year-old multipara and 32-year-old father. The mother's pregnancy was uneventful except for gestational hypertension, for which she took antihypertensives. She had routine prenatal care, including obstetric ultrasounds. The patient was born at term via spontaneous vaginal delivery. Her birth weight was 3.8 kg (75th percentile), length was 52.3 cm (80th percentile). The child had an uneventful postnatal course; corneal opacities were noted bilaterally at birth, and the patient was appropriately referred for ophthalmologic evaluation. The mother said no murmur was auscultated at birth, and the child was discharged on the second day of life. She reportedly passed the newborn hearing screen.

**Figure 1:**
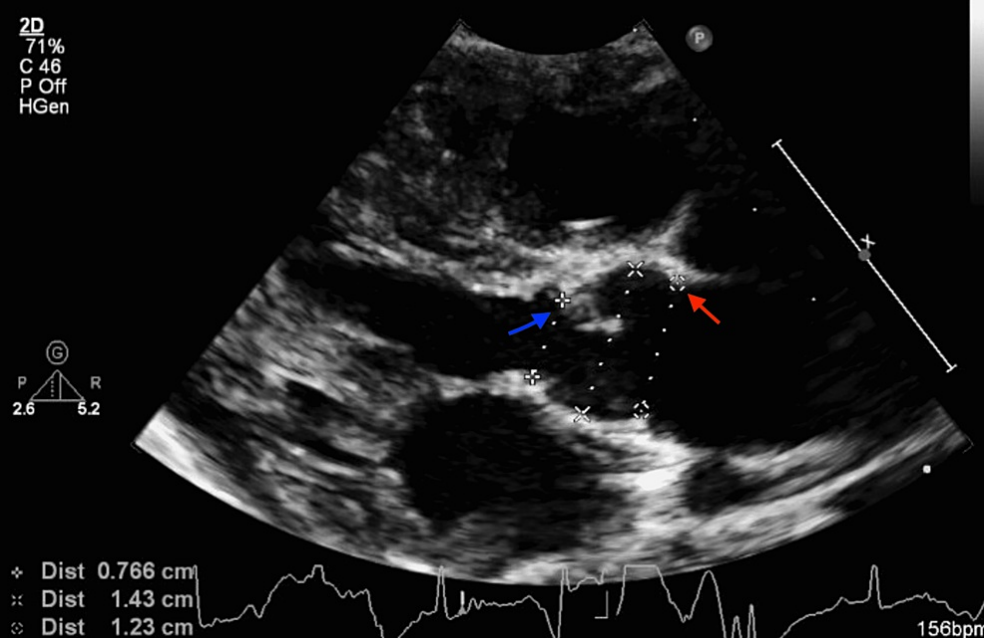
Transthoracic echocardiogram long-axis view showing left ventricular outflow tract, aortic valve area (red arrow), and sub-aortic membrane (blue arrow)

**Figure 2:**
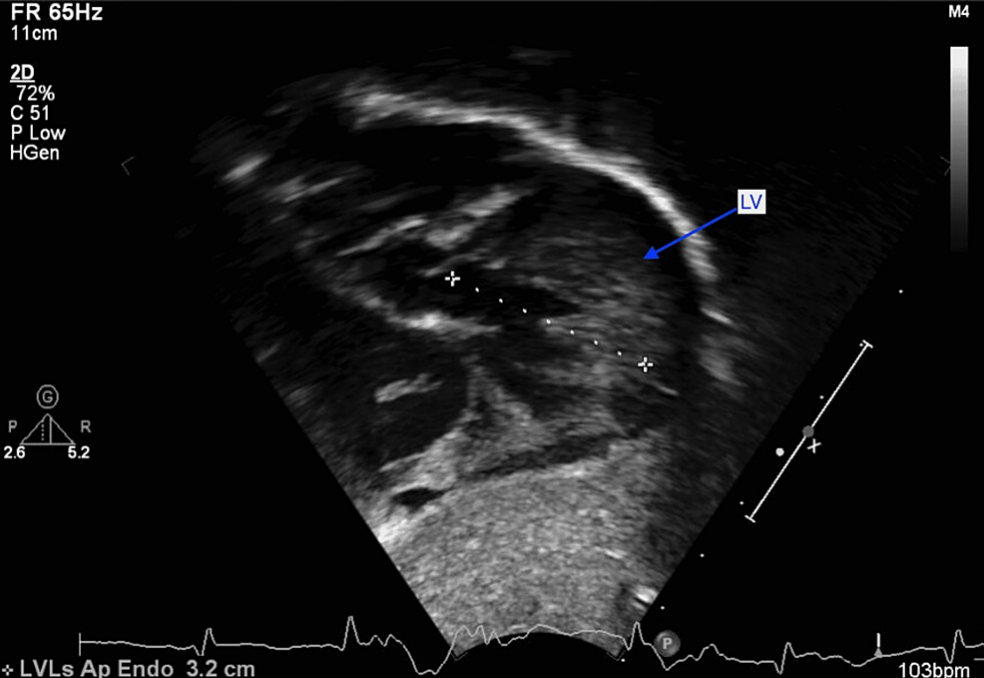
The left ventricle (LV) is moderately dilated with mild hypertrophy. The LV appears to be hypertrabeculated, and measurements of the noncompacted:compacted zones meet criteria (>2:1) for noncompaction

**Figure 3:**
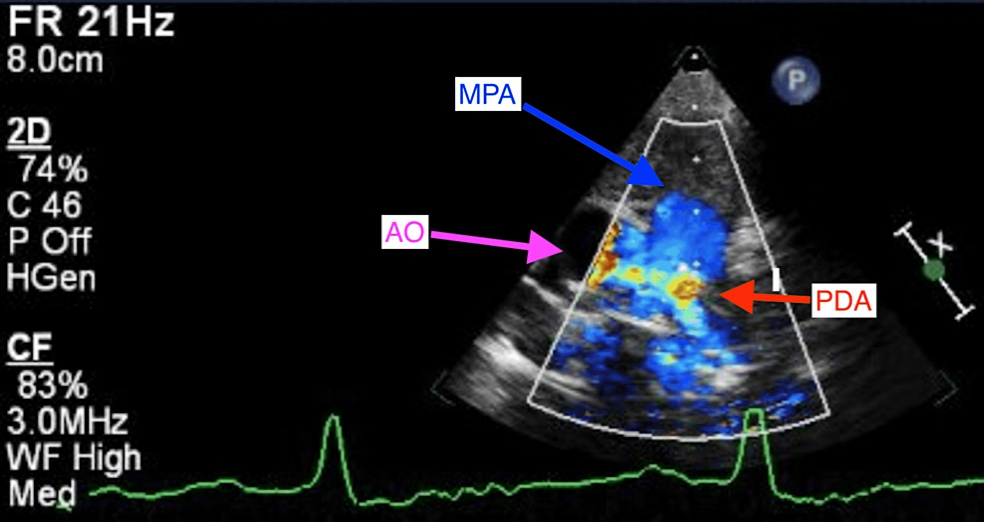
Echocardiography parasternal short-axis view at the aortic valve level with color flow doppler showing turbulent flow of PDA (yellow mosaic signals) with left to right shunting. AO: aorta; MPA:main pulmonary artery; PDA: patent ductus arteriosus

At two weeks of life, on the first visit with her primary care pediatrician, the child was noted to have widely spaced eyes, internal epicanthus, long palpebral fissures, down slanting palpebral fissures, low set posteriorly rotated auricles, flat nasal bridge, long philtrum, thin, cupid bow-shaped upper lip, and a cardiac murmur; therefore, she was sent to be evaluated by a pediatric geneticist and cardiologist. However, she missed all the genetic appointments until the age of nine months. During the first cardiology visit, a harsh 3/6 continuous machine-like murmur was noted in the left infraclavicular region with radiation throughout the precordium and back along with palpable thrill. Echocardiography performed showed subaortic stenosis with the subaortic membrane (Figure 1). There was moderate left ventricular dilatation and hypertrophy (Figure 2). The aortic isthmus was narrow with a diameter of 5.7 mm, and there was a large patent ductus arteriosus (PDA) measuring 4.1 mm with left to right shunting (Figure 3). The echocardiographic findings are presented in Table 1.

**Table 1:** The summary of the echocardiogram PDA: patent ductus arteriosus

Findings	Details
PDA	A left-sided PDA with all left>right shunting, with a peak gradient of at least 39 mm Hg. The PDA minimum diameter: 4.0-4.5 mm.
The aortic valve and left ventricular outflow tract	There appears to be a subaortic membrane, arising mainly in the anterior left ventricular outflow tract.
Mitral valve	Mild mitral valve insufficiency.
Left atrium	Mild-moderate left atrial enlargement.
Left ventricle	The left ventricle is moderately dilated in size and mildly hypertrophied. It appears to be hypertrabeculated, and measurements of the noncompacted:compacted zones meet criteria (>2:1) for noncompaction

The child was seen by an ophthalmologist at two months, and the exam was significant for bilateral opacified cornea, possible media changes as well as damage from glaucoma. The patient was diagnosed with type-1 bilateral Peters Anomaly. 

**Figure 4:**
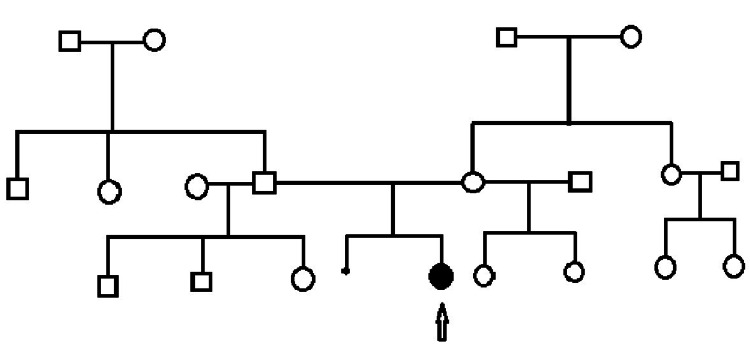
Family pedigree of our patient; the patient is pointed by an arrow

Family history was ascertained. This is a non-consanguineous family of African American (mother) and African American/Native American/Caucasian (father) ancestry. The mother is nearly 35 years old, 164 cm tall, and has a recent diagnosis of hypertension. Dad is 32 years old, 179 cm tall, and healthy. The mother has a 17-year-old daughter and a ten-year-old daughter from a previous union, both healthy and did not have spontaneous pregnancy losses. The father has a 12-year-old son, an 11-year-old son, and an eight-year-old daughter from previous unions. The maternal grandmother has diabetes. The mother has a healthy older sister, the mother of two healthy daughters. The paternal grandmother has diabetes, and the grandfather is well. The father has a healthy brother and a sister, none with children yet. The patient is the first affected in the family. The family pedigree is represented in Figure 4.

At six months of age, the patient was having tachypnea and since the echocardiographic findings suggested left ventricular failure; she was started on furosemide daily therapy and underwent cardiac catheterization. A peak-to-peak invasive systolic gradient of 45mmHg was detected at the level of the membrane in the presence of the large PDA shunt (left-to-right). A 4 mm Amplatzer Piccolo™ Occluder (Abbott Laboratories, Chicago, Illinois, United States) was used to close the PDA with a 50% reduction in the peak-to-peak systolic pressure difference across the membrane. Discharge echocardiography showed no residual PDA and subaortic membrane with a peak systolic gradient of 65 and mean of 42mmHg with mild to moderate left ventricular hypertrophy (LVH) as in Figures 2, 3, 4.

On genetic evaluation at nine months of age, the significant physical findings were as follows: widely spaced eyes, internal epicanthus, long palpebral fissures, everted lower eyelids, down slanting palpebral fissures, bilateral dense corneal opacities, eye malalignment, low-set posteriorly rotated auricles, flat nasal bridge, long philtrum, thin, cupid bow-shaped upper lip, hypodontia, widely spaced nipples, 3/6 systolic murmur, small reducible umbilical hernia, mild rhizomelia of the upper limbs, tapered fingers, single palmar crease on the left, mild 2-3 soft tissue syndactyly, and simple sacral dimple. The child had normal tone, strength, and reflexes in all extremities.

Genetic testing for Peters plus syndrome with *B3GLCT* gene analysis through a diagnostic genetic laboratory was recommended, the result of which came back negative. This significantly decreased the likelihood of Peters plus syndrome as her unifying diagnosis. On the additional panel of ocular genes through the laboratory, the performing lab reported a variant of uncertain significance in the *PAX6* gene c.580G>A p.Gly194Arg. However, *PAX6* genes were shown to cause variable ocular phenotypes, including anterior segment dysgenesis, Peters anomaly, aniridia, and glaucoma [5]. Parental testing results reported the *PAX6* variant was paternally inherited (the same variant of uncertain significance in *the PAX6* gene was noted in the father). High-resolution chromosomal microarray by using array comparative genomic hybridization (aCGH) to examine the DNA extracted from the patient showed a likely pathogenic A copy number gain of 646.6 kb on chromosome 22q11.21(LCR22B-D) of the DiGeorge region (based on coverage in the Database of Genomic Variants) [6]; no extended regions of homozygosity were detected suggestive of uniparental disomy (UPD) or identity by descent (IBD). However, there are 20 genes and transcripts included within the gain, and of these are known Mendelian disease genes (*PI4KA, SERPIND1 (HCF2), SNAP29*, and *LZTR1*). Overlapping gains may be associated with developmental gains and congenital anomalies, including cardiovascular anomalies. This finding was confirmed by fluorescence in situ hybridization (FISH) using a bacterial artificial chromosomes (BAC) probe that hybridized to the chromosome 22q11.21 region (RP11-1058B20; Empire Genomics, Buffalo, New York, United States) and a control probe specific to the subtelomeric region of chromosome 22q (TelVysion 22q, MS607; Abbott Molecular, Chicago, Illinois, United States). Parental genetic testing and chromosomal and microarray analysis were recommended; we offered parental testing for the triplication, and the parents didn't come for the test.

On her most recent follow-up, the patient was 27 months old, and she had undergone uncomplicated cardiac surgery for the subaortic membrane three months earlier. The patient had a history of cough, cold, and wheezing for a day, so she was admitted to the inpatient unit on account of acute asthma exacerbation. She improved with albuterol nebulization first at two hours intervals and later three hours intervals. She was eventually discharged on albuterol every four hours as needed. Her anthropometric measurements at the time of admission were as follows: weight: 15.5 kg (96th percentile), length: 87 cm (40th percentile), and head circumference: 56 cm (>99th percentile). She had normal tone, strength, and reflexes in all extremities. On the latest developmental evaluation, she was found to have a global developmental delay as in Table 3. She could not speak other words except "mama", "dada", and "no". She could not feed with a fork without spilling, could not scribble, imitate activities, stack blocks, or put a block in a cup. She could run, take off clothes, walk up the stairs, and point to her body parts when asked.

**Table 2:** Patient developmental milestones

	Fine/Gross motor	Cognitive	Social	Language
2 months	Appropriate for age (Lifts head off ground)	Delayed (doesn’t follow through visually)	Appropriate for age (social smile)	Appropriate for age (vocalizes)
4 months	Partially delayed (Does not play touching both hands together; but grasps a rattle, supported seating, lifts head to 90 degrees)	Delayed (Doesn’t follow movements by turning head from side to side)	Appropriate for age (laughs out loud)	Appropriate for age (gurgles, coos, babbles)
6 months	Delayed (Does not sit alone, does not lift up the ground in prone position)	Delayed (Doesn’t pass things, doesn’t reach for objects)	Appropriate for age (laughs out loud)	Delayed (no monosyllables)
9 months	Delayed (Does not stand supported, no raking or grabbing movement, does not feed self)	Delayed (Does not pass objects from one hand to another, does not try to find objects after they are removed from view)	Delayed (no stranger anxiety)	Delayed (No disyllables, e.g. “mama” or “dada” sounds)
12 months	Delayed (Does not stand without support, no pincer grasp; but claps hands)	Partially delayed (can bang two things together, but does not follow simple directions)	Delayed (No stranger anxiety, does not play games like “peek-a-boo” or “pat-a-cake”)	Delayed (Does not refer to parents by “mama” or “dada”, no jargon)
18 months	Partially delayed (can drink from a regular cup without spilling, but cannot use cutlery)	Delayed (Does not scribble, does not put blocks in a cup)	Delayed (Does not imitate activities)	Delayed (Does not refer to parents by “mama” or “dada”, no jargon)
24 months	Partially delayed (Runs, can take off clothes, walk up stairs, but does not copy lines and circles or kick a ball)	Delayed (Does not build towers of 4 blocks, does not follow two-step commands)	Delayed (does not imitate adults, no parallel play)	Delayed (Does not speak in two-word sentences, no vocabulary)

In summary, this is a rare case of Peters Anomaly, congenital heart disease including PDA status post (s/p) closure, aortic stenosis pertaining to subaortic membrane s/p repair, macrocephaly, abnormal facial features, and global developmental delay with triplication of 22q11.2 of DiGeorge region and a variant of uncertain significance on genetic evaluation. 

## Discussion

To the best of our knowledge, we are presenting the third reported case of triplication of the 22q11.2 chromosomal region. This triplication has a broad range of phenotypes affecting phenotypic and cognitive development, comparable to the two reported cases in the literature. The first patient presented with speech delay, learning disabilities, and dysmorphic features [4]. The second patient had an aggravated phenotypic feature characterized by facial dysmorphisms, heart defects (restrictive VSD and membranous subaortic stenosis), and urogenital malformations; the same patient was described in two separate articles [3,7]. Table 3 shows the comparison between the current case and the triplication cases in the previously published case reports.

**Table 3:** The comparison between the current case and the triplication cases in previously published case reports

Clinical characteristics	Yobb et al., 2005 [4]	Vaz et al., 2015 [3]	Current case
Age at evaluation	8 years	20 years	28 months
22q11.2	Triplication	Triplication	Triplication
Heart defect	-	+	+
Velopharyngeal insufficiency	-	+	-
Palatal defect	-	-	-
Hearing impairment	+	+	-
Failure to thrive	-	-	-
Sleep apnea	-	+	-
Urogenital abnormality	+	-	-
Cognitive defects	+	-	-
Psychiatric disorder	+	N/A	N/A
Behavioral problems	N/A	+	-
Developmental delay	N/A	N/A	+
Seizures	-	-	-
Headache	+	N/A	N/A
Mild rhizomelia	N/A	N/A	+
Tapered fingers	N/A	N/A	+
Single palmar crease (left hand)	N/A	N/A	+
Microcephaly	-	N/A	-
Long narrow face	+	-	-
Prominent forehead	-	N/A	-
Hypertelorism	+	N/A	+
Epicanthal fold	+	+	+
Upslanting palpebral fissures	-	N/A	+
Downslanting palpebral fissures	-	N/A	-
Strabismus	+	N/A	N/A
Myopia	+	N/A	N/A
Corneal opacity	N/A	N/A	+
Broad nasal bridge	N/A	-	+
Prominent nose	N/A	+	-
Long philtrum	N/A	+	+
Smooth philtrum	N/A	+	-
Thin, cupid, bow-shaped upper lip	N/A	-	+
Low set auricles	N/A	-	+
Dysplastic ears	N/A	-	-
Preauricular fistula	N/A	-	-
Micrognathia	N/A	-	-
Retrognathia	N/A	+	-
Supernumerary teeth	N/A	-	-
Dental cavities	N/A	+	-
Absent dentition	N/A	N/A	+
Recurrent infections	N/A	+	+
Dermatological abnormalities	N/A	+	-
Allergies	N/A	+	-
Widely spaced nipples	N/A	-	+
Umbilical hernia	N/A	-	+

Central corneal opacification can lead to delayed visual development progression caused by defects in the Descemet membrane and the posterior stroma [8]. Our patient has Peters anomaly, a rare, congenital eye malformation involving the anterior segment of the eye characterized by an opaque cornea and blurred vision. Multiple oculars and systemic malformations have been observed with this anomaly, and novel comorbidities continue to be reported.

We thus present Peters anomaly as a new characteristic of triplication of the 22q11.2 chromosome region. This was noted in our patient with a 646 Kb triplication of 22q11.2 (LCR22B-D) of the DiGeorge region with a different eye anomaly, consistent with a phenotypic expansion that has not been detected been reported in the literature. Although it is rare, we reaffirm that medical practitioners should take heed to the similarities between 22q11.2 triplication, microduplication, and deletion syndromes to prevent misdiagnosis. in addition, these syndromes should be taken into account in a patient presenting with a dysmorphic face associated with heart defects, developmental delay, and other malformations.

Increased dosage of this region remains unclear whether it causes a recognizable phenotype. A clinically significant phenotype has been theorized that could be a benign polymorphism or risk variant requiring a "second hit." Furthermore, a second hit could be an additional copy of the same region, assuming that one or more involved genes are dosage sensitive. It is suggested that an increased dosage of these genes might increase the risk for cardiac abnormalities [9].

## Conclusions

We described in depth the phenotypic features of a female with triplication of the 22q11.2 DiGeorge region to highlight the consequence of increased dosage number of the 22q11.2chromosomal region. Moreover, this patient's phenotypic appearance may support a new feature and characteristic of triplication of this genomic region, which includes phenotype influencing cardiovascular development, facial appearance, cognition, and developmental. We present Peters anomaly as a new characteristic of triplication of the 22q11.2 chromosome region.

## Appendices

Online Mendelian Inheritance in Man (OMIM) number for syndromes

Peters Anomaly (OMIM#604229)

Peters plus syndrome (OMIM#261540)

DiGeorge syndrome (OMIM#188400)

Velocardiofacial syndrome (#192430)

Chromosome 22q11.2 microduplication syndrome (OMIM#608363)

Chromosome 22q11.2 deletion syndrome (OMIM#611867)

## References

[1] Ryan AK, Goodship JA, Wilson DI (1997). Spectrum of clinical features associated with interstitial chromosome 22q11 deletions: a European collaborative study. J Med Genet.

[2] Robin NH, Shprintzen RJ (2005). Defining the clinical spectrum of deletion 22q11.2. J Pediatr.

[3] Vaz SO, Pires R, Pires LM, Carreira IM, Anjos R, Maciel P, Mota-Vieira L (2015). A unique phenotype in a patient with a rare triplication of the 22q11.2 region and new clinical insights of the 22q11.2 microduplication syndrome: a report of two cases. BMC Pediatr.

[4] Yobb TM, Somerville MJ, Willatt L (2005). Microduplication and triplication of 22q11.2: a highly variable syndrome. Am J Hum Genet.

[5] Nishina S, Kohsaka S, Yamaguchi Y, Handa H, Kawakami A, Fujisawa H, Azuma N (1999). PAX6 expression in the developing human eye. Br J Ophthalmol.

[6] (2022). Database of Genomic Variants. http://dgv.tcag.ca/dgv/app/links?ref=GRCh37/hg19.

[7] Pires R, Pires LM, Vaz SO (2014). Screening of copy number variants in the 22q11.2 region of congenital heart disease patients from the São Miguel Island, Azores, revealed the second patient with a triplication. BMC Genet.

[8] Salik I, Gupta A, Tara A, Zaidman G, Barst S (2020). Peters anomaly: a 5-year experience. Paediatr Anaesth.

[9] Dale B, Modi BM, Jilderda S (2017). Atypical autism in a boy with double duplication of 22q11.2: implications of increasing dosage. NPJ Genom Med.

